# National Health-Oriented Hazard Assessment in Iran Based on the First Priority for Action in Sendai Framework for Disaster Risk Reduction 2015–2030

**DOI:** 10.1155/2021/5593223

**Published:** 2021-09-28

**Authors:** Hamidreza Khankeh, Yousof Akbari Shahrestanaki, Saiedeh Bahrampouri, Mehdi Beyramijam

**Affiliations:** ^1^Health in Emergency and Disaster Research Center, The University of Social Welfare and Rehabilitation Sciences, Tehran, Iran; ^2^Department of Clinical Science and Education, Karolinska Institutet, Stockholm, Sweden; ^3^Department of Pre‐Hospital Medical Emergencies, School of Paramedical, Qazvin University of Medical Sciences, Qazvin, Iran; ^4^Hamadan University of Medical Sciences, Hamadan, Iran

## Abstract

**Background:**

Understanding disaster risk is the first priority for action based on the Sendai Framework for Disaster Risk Reduction 2015–2030 (SFDRR), and hazard assessment is the first step in the assessment of disaster risks. Therefore, assessing health-oriented hazards is the first measure in disaster risk assessment in the medical universities area in Iran. This article introduces a national experience and results obtained from designing a national tool for defining and assessment of health-oriented hazards in Iran.

**Methods:**

In the present study, a National Health-Oriented Hazard Assessment tool (NHHAT) was developed by experts and implemented by the Iranian Ministry of Health for gathering data according to frequency, probability, magnitude, and vulnerability of the hazards to identify the first ten hazards of medical universities in the two decades ago (2000–2021). Finally, the top 20 health-oriented hazards were identified among the ten hazards reported by each university.

**Results:**

According to the findings, the four most important hazards were road traffic accidents, earthquakes, drought, and seasonal floods. Nevertheless, the hazards such as desertification, tunnel events, soil liquefaction, mass population movement, and sea progression were among the rarest ones reported in the medical universities in Iran.

**Conclusion:**

Many functional aspects of disaster risk management depend on the realistic and accurate information related to the main elements of risk, especially the probable hazards in the communities. The comprehensive hazard assessment can only provide such information using context-bond tools. This is an applied study and a national implementation to fulfill the priority of the Sendai framework (i.e., understanding disasters risk) in Iran. It is suggested that other countries should also compile standard tools to explore the hazards for designing up-to-date hazard maps.

## 1. Background

As mentioned in the Sendai Framework for Disaster Risk Reduction (2015–2030), all policies and plans for disaster risk management should be based on understanding disaster risk from all aspects, including the vulnerability of individuals and properties, capacities, exposures, and characteristics of the environment and specifically the hazard behavior. In the past 10 years to 2015, 700 thousand people have lost their lives, more than 1.4 million injured, and about 23 million become homeless because of disasters all over the world [1].

Evidence suggested that the exposure of individuals and properties to hazards had high and fast growth in all countries in comparison to reduce their vulnerability. Therefore, new risks and increased damages caused by disasters at local and national levels have been brought up [2]. Natural hazards in Asia caused 90% of the affected population, 50% of deaths, and economic damage in the world [3]. In 2014, 202 natural disasters were recorded in Asia alone, in which 10,107,000 people were injured and 87,760,054 were affected [4]. Meanwhile, more than 90% of all casualties from natural disasters happened in developing countries [5–7].

Iran is in a region prone to many natural and manmade hazards. Hazards such as earthquakes, drought, and floods are the most important causes of death and economic damages [8]. During the previous three decades, many disasters have taken place, such as Rudbar–Manjil earthquake (1990), Bam earthquake (2003), Golestan floods (2000 and 2005), Azerbaijan earthquake (2012), Bushehr earthquake (2013), and Kermanshah earthquake (2017) [9], in which more than 109,000 people died and 150,000 were injured [8]. The most important features that change a hazard into a disaster are its likelihood of occurrence, vulnerability, and the response capacity of the affected community. Not all hazards lead to a disaster but only those whose impacts and outcomes are beyond the capacity of the affected area are considered disasters [5].

Regarding the international documents (the Hyogo and Sendai frameworks), the new approach in world disaster management is to decrease the disaster risks. Then, it is necessary to pursue the following objectives: preventing new disaster events and reducing the impacts of the existing ones. This will be possible only through implementing integrated and inclusive economic, structural, legal, social, health, cultural, educational, environmental, technical, political, and organizational measures. This can lead to the prevention and reduce the exposure to the hazards and the damages caused by disasters. It also promotes the preparedness for responding and recovering, which may result in the enforcement of resilience. The most important prerequisite to achieve the above goals is to understand disaster risk at local, regional, national, and universal levels. Understanding the disaster risk and risk assessment process begins with the technical hazard assessment by analyzing the probable hazards of the region [10].

Hazard assessment is a process to understand the behavior of the hazard including its frequency, probability, severity, and impact that threatens the community [5]. Hazard assessment answers the following questions: Which hazards are plausible in the community? How probable are they? How much will be the damages and their losses? What will be their impact on the community? and How much the community is vulnerable to the hazards [11]? Hazard assessment is necessary according to the unique context of each community/country (climatic, cultural, social and economic, geographic, housing patterns, and political sustainability) [5]. Therefore, it is essential to design and develop a standard analysis tool compatible with cultural and contextual factors.

In a short review of the literature, there are different hazard assessment methods and tools. Each of them used a different method to identify and prioritize the hazards. The hazard assessment tools are generally divided into two major groups: quantitative and qualitative.

For example, Threat and Hazard Identification and Risk Assessment (THIRA) is a quantitative tool that was produced and suggested by the Federal Emergency Management Agency (FEMA) in the United States of America [12].

Based on our knowledge, there is no comprehensive hazard assessment tool in national and in the health area adapted to Iranian cultural and contextual conditions and no reliable studies related to hazard analysis of the country. The research team of Health in Emergency and Disaster at the University of Social Welfare and Rehabilitation Sciences conducted the present study with two goals. The first goal was to design and develop a national tool to assess the health-oriented hazards. The second goal was to explore and identify the country's hazard list based on the geographic areas managed by medical and healthcare universities, considering the indicators that have influenced the rating of the hazards. This article is intended to introduce the “context-bond HHAT” which has been produced based on the national and international experiences that resulted in identifying the probable hazards in the whole country.

## 2. Methods

The present study was conducted in Iran during the years 2017–2018. The data relating to possible hazards and their impacts (the items in Tables 1–4) were gathered from the medical universities area to identify the first ten hazards in each medical university during the years 2000–2017. Accordingly, an NHHAT was designed to extract and evaluate hazards based on their behavior (probability, frequency, vulnerability, and magnitude) and geographical characteristics. This tool was developed at the Health in Emergencies and Disaster Research Center, University of Social Welfare and Rehabilitation Sciences in Tehran, and then approved by the Iranian Ministry of Health and Medical Education. The criteria and thresholds in this tool were prepared according to the national and international experiences and developed based on the opinion of experts in the field of emergency and disaster, using valid literature and the available hazard assessment tools. The experts include 2 emergency medicine specialists, 3 health in emergency and disaster specialists, 1 disaster epidemiologist, 1 prehospital emergency specialist, 1 geologist and seismologist, and 1 meteorologist. After approval by the health system authorities, the tool was introduced by the Ministry of Health and Medical Education and administered by all parts of the health system around the country.

For designing this tool, first, a list of 53 different types of hazards (natural, technologic, biological, chemical, and radiological) was identified and placed in the first column of the checklist. This list was open-ended, so that more hazards could be added later. The information used to define this list was provided in the District Disaster Management Organization and the other related organizations such as Agriculture Jihad Organization, Meteorological Organization, University of Tehran Institute of Geophysics, Red Crescent Society, Fire Departments, local trustees, and reliable historical documents. In the next, the four essential criteria include probability, frequency, vulnerability, and magnitude (that each has different constant coefficients), and also, different items to determine and score them (Tables 1–4) were used for scoring and ranking the first ten hazards (Table 5) in each university during the years (2000–2017). It should be noted that the constant coefficients are determined based on expert opinion. This period was chosen based on the feasibility and availability of valid and reliable data. The reliability and availability of information are derived from the Iran Development Outlook Document (in 20 years). All Iranian organizations have defined their development strategies according to this 20-year timeline. Also, it should be noted that as access to the physical, economical, social, environmental, and other information of variables related to vulnerability was not possible, the variable of “exposure to damage” was used to determining the vulnerability. To the rank of the identified hazards (in each university), the score of each criterion (based on the guides in Tables 1–4) is multiplied by its constant coefficient by the stakeholders who were supposed to complete the tool, and the sum of the obtained scores creates the final hazard score (i.e., the total hazard score = [frequencies (1−5) *x* 7] + [probability(1−2) *x* 2] + [magnitude(1−6) *x* 6 ] + [vulnerability(1−5) *x* 5]). Then, the hazards were arranged according to their highest scores, and the first ten hazards were determined in each university.

To complete the tool, guidance and a definition of the related terms were added as tool guidance and published in the book “national tools for assessing health in emergency and disaster” and later became available for all medical universities all over the country [13]. To increase the accuracy of data collection, the Secretariat of the National Working Group on Health in Emergency and Disasters in the Iranian Ministry of Health and Medical Education arranged some workshops and training courses to introduce the tool for all stakeholders to increase the accuracy of data collection.

The training included introducing the basic concepts related to disasters and the way to fill the tool. All the data (the first ten hazards identified in each university based on Table 5) were entered in Excel and analyzed. Finally, the first 20 hazards were selected and ranked according to the number of universities where each hazard was reported. For example, the first hazard (i.e., the road traffic accidents) is the hazard that was identified and reported by more universities.

## 3. Results

**Figure 1:**
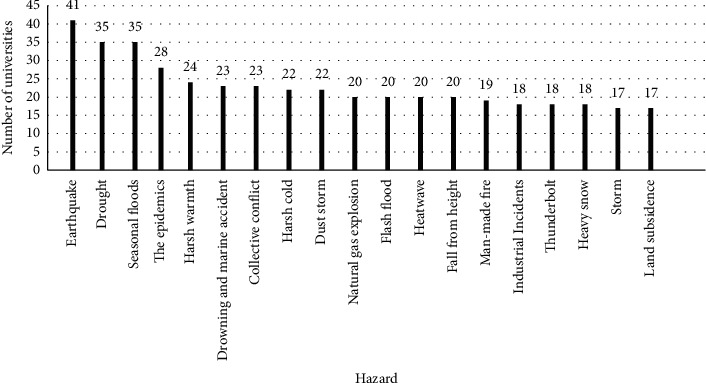
The top 20 health-oriented hazards in Iran based on data from years 2000–2017.

After designing the NHHAT and gathering data, the hazards and their occurrences (frequency), probability, magnitude, and impacts (number of injured or killed people, financial impact, and other data based on Tables 1–4) from 45 medical universities were analyzed to extract a list of the top 20 health-oriented hazards in Iran. Data analysis showed that “road traffic accidents” were the first priority for the Iranian Health System. Also, earthquakes, droughts, and floods are the three hazards that ranked second to fifth. The hazards 6–20 are shown in Figure 1. Except for the top 20 hazards which were very common, some rare hazards, such as desertification, tunnel events, soil liquefaction, mass population movement, and sea progression, were explored for the first time, which based on our knowledge was not ranked or reported before in the other studies. These hazards and their frequencies are given in Table 6.

## 4. Discussion

Although gathering data with quantitative hazard assessment tools is more difficult in comparison to the qualitative tools, their precision is much higher [14]. In this study, the local hazard assessment tool with a quantitative approach has been used to determine and extract hazards using the data of the regions covered by the universities of medical sciences from all over the country. Also, in this study, similar to the health hazard assessment and prioritizing method (hHAP) which was used by the Los Angeles County Department of Public Health, an open-ended hazard checklist was used to determine the hazards. In this tool, a list containing 36 probable hazards out of 60 identified ones was chosen and reviewed [15]. However, in the Federal Emergency Management Agency (FEMA) and Coppola recommended method, the possible hazards are identified using different sources mentioned in the list. For example, in this way, a list of hazards of the examined area is extracted by using methods such as brainstorming, historical research studies in media archives, governmental documents, the collective memory of citizens, taking an overview of existing plans and programs, using maps, and follow-up interviews, and then choosing and listing hazards based on priorities [5, 12].

In this study, the criteria such as “frequency,” “magnitude,” “vulnerability,” and “hazard probability” have been used to score and prioritize the hazards in about 20 years. However, this interval is not the same for all tools and methods. In a few cases, no time interval is considered at all. For example, in the hazard assessment tool by the Center for the Public Health and Disasters at the University of California, Los Angeles (UCLA) [16], and hHAP [15] tools have considered 25 years (because this interval is necessary to witness some rare hazards). However, the FEMA and Coppola methods have not considered any time interval for collecting data [5, 12]. The Emergency Management Ontario Ministry of Community Safety and Correctional Services, 2012, has defined a definite time interval to overview the number of each hazard happenings [17]. Different methods are used to rank hazards in the hazard assessment tools and methods. In most of the methods, estimation of the “risk score” for each hazard has been used to rank and determine the importance. For example, in the Coppola method [5], Kaiser Permanente Hazard Vulnerability Analysis (HVA) tool [18], hHAP tool [15], and UCLA tool [18] used risk scores for each hazard to prioritize them. Each of these tools and patterns used different indicators to estimate risk scores and finally rank the hazards. In the Coppola model, indicators related to each hazard such as “frequency,” “likelihood,” “magnitude,” “location of hazard occurrence,” “estimated spatial extent of hazard impact,” “duration of hazard event,” “speed of hazard onset,” “availability of hazard warnings,” and “time-based patterns of the hazard” are used. In this pattern, background information of the community such as information on geographic environment, assets and infrastructure facilities, demographic features of the population, vulnerabilities, and responding capacities are also used to estimate the risk of each hazard [5]. Hazard ranking factors in FEMA are “likelihood of hazard occurrence” and “the importance of hazard impact” [12, 15], and both of them are the key criteria used in most of the hazard assessment tools and hazard assessment methods, and they have been used in our tool too [5, 12, 19].

In the present study, the criteria of “probability” with the constant coefficient [7] have been used first. But after some experience, it was concluded to reduce them to 2 instead. Since probability/likelihood of hazard occurrence is multifactorial and depends on changes in the present and future, there is a need to have accurate scientific studies to collect detailed information which is not possible now. The impacts of hazards are unique to every part of the country with many possible quantitative features [12]. Therefore, it is used based on the information obtained from the two criteria of hazard, i.e., “magnitude” and “vulnerability,” to estimate the impact of the hazard. The “size of the affected geographic area,” “number of displaced households,” “number of fatalities,” “number of injuries and illnesses,” and “disruption to critical infrastructures” are the factors required to estimate the hazard impacts in the FEMA pattern [12]. In the hHAP tool, the impact of the hazard on “community,” “public health system,” “medical system,” and “psychological health” are considered [15]. In the present study, 60 hazards were identified in the regions covered by the university of medical sciences (this includes the whole country to some extent); among which, the first 20 hazards were selected and reported based on their priorities. Among these 20 hazards, the first 5 were “traffic accidents,” “earthquakes,” “drought,” “floods,” and “epidemics,” and the final three ones were “heavy snow,” “storms,” and “land subsiding.”

Though many sources have reported 31 kinds of hazards in Iran [20–22], our study explored 60 different kinds of hazards including natural and manmade which needed to be considered. The results obtained by the Emergency Events Database (EM-DAT) [23] emphasized the priority and importance of earthquakes in Iran in comparison to other hazards. Based on the EM-DAT results, earthquakes had the most number of occurrences among other natural hazards during 1900–2016. Though the success of many hazard assessment tools and models depends on reliable information obtained from the community considering contextual/cultural factors, the approach of this study was to use a context-bond tool as a national tool with highly accurate information in the country covered by universities all over the country. It is very rare to find a tool among all the existing tools and methods with such comprehensive coverage. Hence, the tool was first used in a pilot study at the beginning of 2018 and then developed into hazard assessment software.

The experience of using this tool showed that the national tool used in this study was an achievement in comparison to other tools, and despite its new perspective, it was successful enough in extracting the health hazards of the country. However, it seems there are some problems in estimating the likelihood/probability of hazard. It is, therefore, suggested that other researchers look for a way to solve this problem and update the tool to get the first step in disaster risk management, which is understanding disaster risk. It is finally recommended to quantify the hazard assessment tools to provide more credible information.

## 5. Conclusion

In the present study, a health-oriented hazard assessment tool was developed with the support of the national government. Many functional aspects of disaster risk management depend on realistic and trustworthy information related to the hazard and its components in the target area. It is, therefore, necessary to assess the hazards by using national tools and valid scientific methods to make them available at all different levels for those involved in disaster risk management in the country. These tools need to be continually updated, and more valid information is needed for producing credible and strong scientific evidence to plan for country risk management. On the other hand, since it is difficult to predict the probability of hazards according to continuous changes of infrastructures and population characteristics and the multifactorial nature of this important component of risk, it is recommended to have more studies in the future to find the influencing factors on the likelihood of hazards. Urbanization, technological development, development, and climatic changes have subjected human beings to be exposed to more technological hazards and urban events, which introduce the need for advanced scientific studies in this field.

## Figures and Tables

**Table 1:** Ranking the hazard according to its probability.

Probability	Definition
1	The occurrence probability of the hazard is very weak
2	The hazard is likely to occur over the next 20 years
3	The hazard is likely to occur in the next 10–19 years
4	The hazard is likely to occur in the next 5–9 years
5	The hazard is likely to occur in less than 5 years

**Table 2:** Ranking the hazard according to its frequency.

Rank of frequency	Definition
1	None in the last 20 years
2	Once in the last 20 years
3	Two or three times in the last 20 years
4	Four or five times in the last 20 years
5	More than five times in the last 20 years

**Table 3:** Ranking the hazards according to its magnitude.

Rank of magnitude	Definition
1	(i) No impact on human health
	(ii) Property damage less than 25000$
	(iii) No homeless or displacement
	(iv) No impact on health services

2	(i) 1 or 2 killed
	(ii) 1–4 injured
	(iii) Property damage from 25000 to 250000$
	(iv) 1–100 homeless/displaced
	(v) 0–2 hours disruption in health services

3	(i) 3–5 killed
	(ii) 5–9 injured
	(iii) Property damage from 250000$ to 2.5 million$
	(iv) 101–1000 homeless/displaced
	(v) 2–12 hours disruption in health services

4	(i) 6–9 killed
	(ii) 10–99 injured
	(iii) Property damage from 2.5 million$ to 25 million$
	(iv) 1001–10000 homeless
	(v) 12–24 hours disruption in health services

5	(i) More than 10 killed
	(ii) More than 100 injured
	(iii) Property damage more than 25 million$
	(iv) More than 10000 homeless
	(v) More than 24 hours disruption in health services

**Table 4:** Ranking the level of vulnerability.

The rank of exposure to damage	Definition
1	Less than 20% of the population at risk of health, financial, and functional damage
2	20–40% of the population at risk of health, financial, and functional damage
3	40–60% of the population at risk of health, financial, and functional damage
4	60–80% of the population at risk of health, financial, and functional damage
5	80–100% of the population at risk of health, financial, and functional damage

**Table 5:** Table of identifying the top ten hazards in each medical university.

Hazard	Frequency (7)	Likelihood/probability (2)	Magnitude (6)	Vulnerability (5)	Hazard total score
Hazard 1, 2, 3,…, 10	… *x* 7 = …	… *x* 2 = …	… *x* 6 = …	… *x* 5 = …	—

**Table 6:** The hazards that were explored for the first time in the medical universities area in Iran.

Row	Hazard	Number of universities
1	Water uplift	15
2	Falling	14
3	Soil liquefaction	13
4	Ice storm	11
5	Water pollution	9
6	Mass gathering	7
7	Insects and wild animals attack	6
8	Tunnel incident	5
9	Mountain incident	4
10	Events during project implementation	4
11	Sea level rise	4
12	Plant pests	3
13	Desertification	—
14	Sea oil pollution	3
15	Sea waves flows	2
16	Bridge collapse	2
17	Deforestation	1
18	Sea events	1

## Data Availability

The datasets used and/or analyzed during the current study are available from the corresponding author upon request.
